# Optimal size of pterygium excision for limbal conjunctival autograft using fibrin glue in primary pterygia

**DOI:** 10.1186/s12886-018-0790-6

**Published:** 2018-06-07

**Authors:** Ho Sik Hwang, Kyong Jin Cho, Gabriel Rand, Roy S. Chuck, Ji Won Kwon

**Affiliations:** 1 Department of Ophthalmology, Chuncheon Sacred Heart Hospital, Hallym University, Chuncheon, Korea; 2 Department of Ophthalmology, Dankook University College of Medicine, Cheonan, Korea; 3 Department of Ophthalmology, Montefiore Medical Center, Bronx, New York, USA; 4 Department of Ophthalmology, Myongji Hospital, Seonam University College of Medicine, 55 Hwasu-Ro 14, Deokyang-Gu, Goyang-Si, Gyeonggi-Do 10475 Korea

**Keywords:** Primary pterygium, Excision size, Conjunctival autograft, Mitomycin C, Fibrin adhesion

## Abstract

**Background:**

In our study we describe a method that optimizes size of excision and autografting for primary pterygia along with the use of intraoperative MMC and fibrin glue. Our objective is to propose a simple, optimizedpterygium surgical technique with excellent aesthetic outcomes and low rates of recurrence and otheradverse events.

**Methods:**

Retrospective chart review of 78 consecutive patients with stage III primary pterygia who underwent an optimal excision technique by three experienced surgeons. The technique consisted of removal of the pterygium head, excision of the pterygium body and Tenon’s layer limited in proportion to the length of the head, application of intraoperative mitomycin C to the defect, harvest of superior bulbar limbal conjunctival graft, adherence of graft with fibrin glue. Outcomes included operative time, follow up period, pterygium recurrence, occurrences of incorrectly sized grafts, and other complications.

**Results:**

All patients were followed up for more than a year. Of the 78 patients, there were 2 cases of pterygium recurrence (2.6%). There was one case of wound dehiscence secondary to small-sized donor conjunctivaand one case of over-sized donor conjunctiva, neither of which required surgical correction. There were no toxic complications associated with the use of mitomycin C.

**Conclusion:**

Correlating the excision of the pterygium body and underlying Tenon’s layer to the length of the pterygium head, along with the use intraoperative mitomycin C, limbal conjunctival autografting, and fibrin adhesionresulted in excellent outcomes with a low rate of recurrence for primary pterygia.

## Background

Pterygium is one of the most common ocular surface diseases with a global pooled prevalence calculated at 10.2% [1]. Pterygium surgery has evolved over last 50 years with the aim of reducing post-surgical complications, primary of which is recurrence. A 2016 Cochrane meta-analysis examined the two most currently popular techniques, limbal conjunctival autografting and amniotic membrane grafting, and found conjunctival autografting superior with respect to lower 6 month recurrence rates [2]. Surgeons make use of variations of conjunctival autografting in an effort to further reduce recurrence rates. A 2011 meta-analysis found conjunctival autografting with fibrin glue superior to sutures with respect to lower recurrence rates [3]. In addition, there have been a number of randomized control studies showing lower recurrence rates with the adjuvant use of intraoperative mitomycin C (MMC) [4–6]. In our practice we routinely make use of limbal conjunctival autografting with intraoperative MMC and fibrin glue.

The contribution of our study is that we introduce a technique that standardizes the size of excision and grafting as a function of the length of the pterygium head. The extent of excision necessary to prevent recurrence is still a matter of debate, with a spectrum of opinions ranging from simple avulsion and removal of the pterygium head to extensive subconjunctival dissection and excision of the entire pterygium to the point of insertion [7–12]. We developed this technique because we believe that the size of excision and graft for primary pterygia should be a continuous function based on the tissue’s aggressiveness as measured by the size of the pterygium head.

In our study we describe a method that optimizes size of excision and autografting for primary pterygia along with the use of intraoperative MMC and fibrin glue. Our objective is to propose a simple, optimized pterygium surgical technique with excellent aesthetic outcomes and low rates of recurrence and other adverse events.

## Methods

This study is a retrospective chart review of patients having conjunctival autograft surgery for primary pterygia. We included 78 consecutive patients with primary nasal pterygia and no previous ocular surgeries or active ocular diseases. Included patients had initial visits in our hospitals from January 2014 to August 2015. This is a multicenter study performed by three experienced surgeons, the first in Myongji Hospital in Seonam University (56 eyes), the second in Dankook University Hospital (20 eyes), and the third in Chuncheon Sacred Heart Hospital in Hallym University (2 eyes). All surgeons followed an identical, predetermined surgical protocol. The presurgical factors we accounted for include age, gender, and the length of pterygium head. All pterygia were stage III, the pterygium head between the limbus and the pupillary margin [13].

The primary outcome of this study was pterygium recurrence. Recurrence was defined as any postoperative regrowth of fibrovascular tissue crossing the corneoscleral limbus onto the clear cornea. The secondary outcome of this study was the sizing of the graft as measured by rates of wound dehiscence or oversized grafts. Other reported results include the operation time and other postoperative complications. The data was analyzed using basic statistics including percentages, ranges, means, and standard deviations. Mean values are reported with ±1 standard deviation. We adhered to the tenets of the Declaration of Helsinki. Appropriate Institutional Review Board/Ethics Committee approvals were obtained from IRB of Chuncheon Sacred Heart Hospital, Dankook University Hospital and Myongji Hospital and the need for consent was waived for this retrospective chart review study by them.

### Surgical procedure

**Fig. 1:**
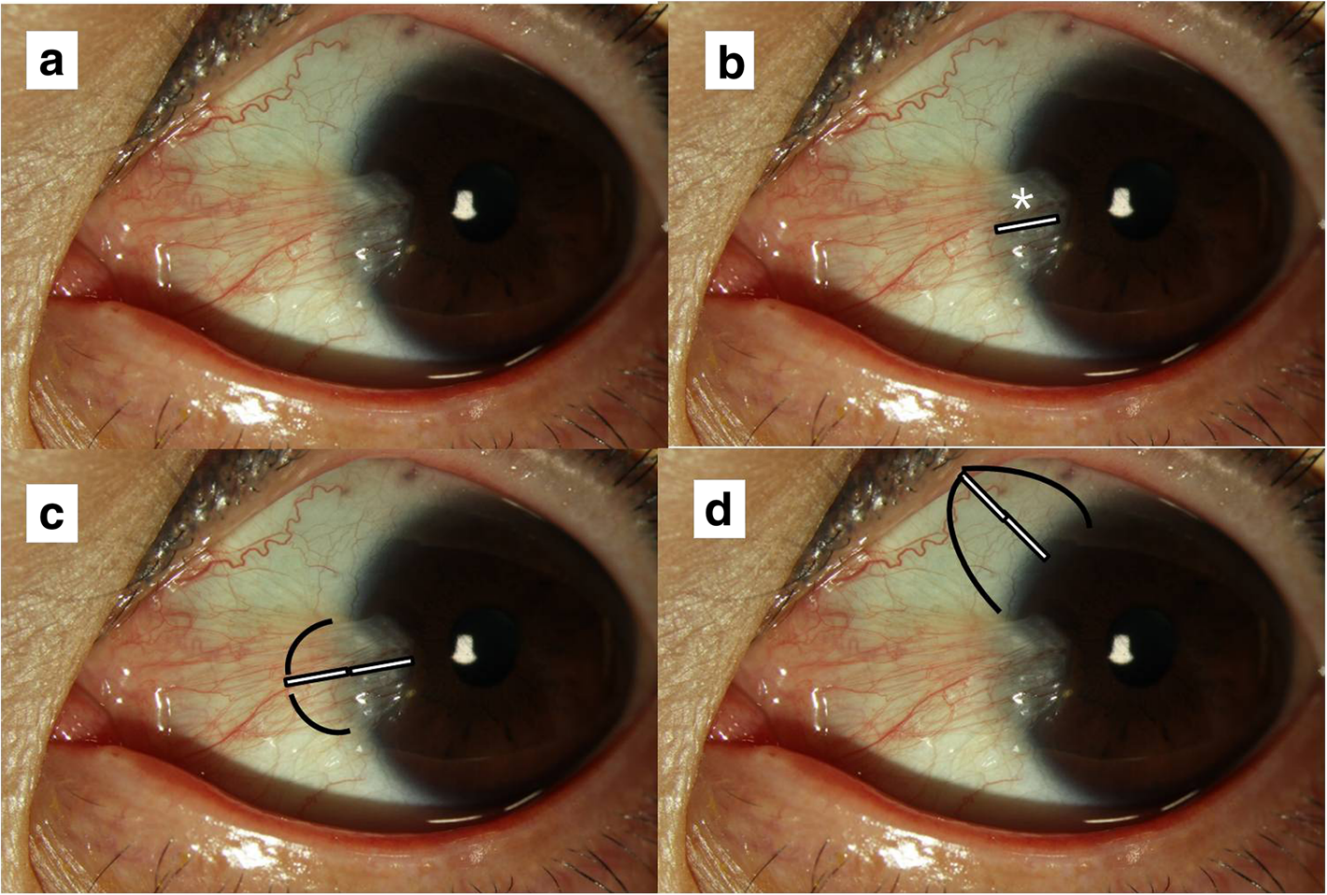
The preoperative appearance of a primary pterygium (**a**). The length from the limbus to the apex of pterygium head (*) was measured (**b**). Following the same line, a point indicating the previously measured distance (*) from the limbus onto the pterygium body was marked (**c**). From this point, two symmetric curvilinear lines were marked from the point to the superior and inferior margins of the pterygium at the level of the limbus (**c**). Autograft size on the superior bulbar conjunctiva were measured and marked out (**d**)

**Fig. 2:**
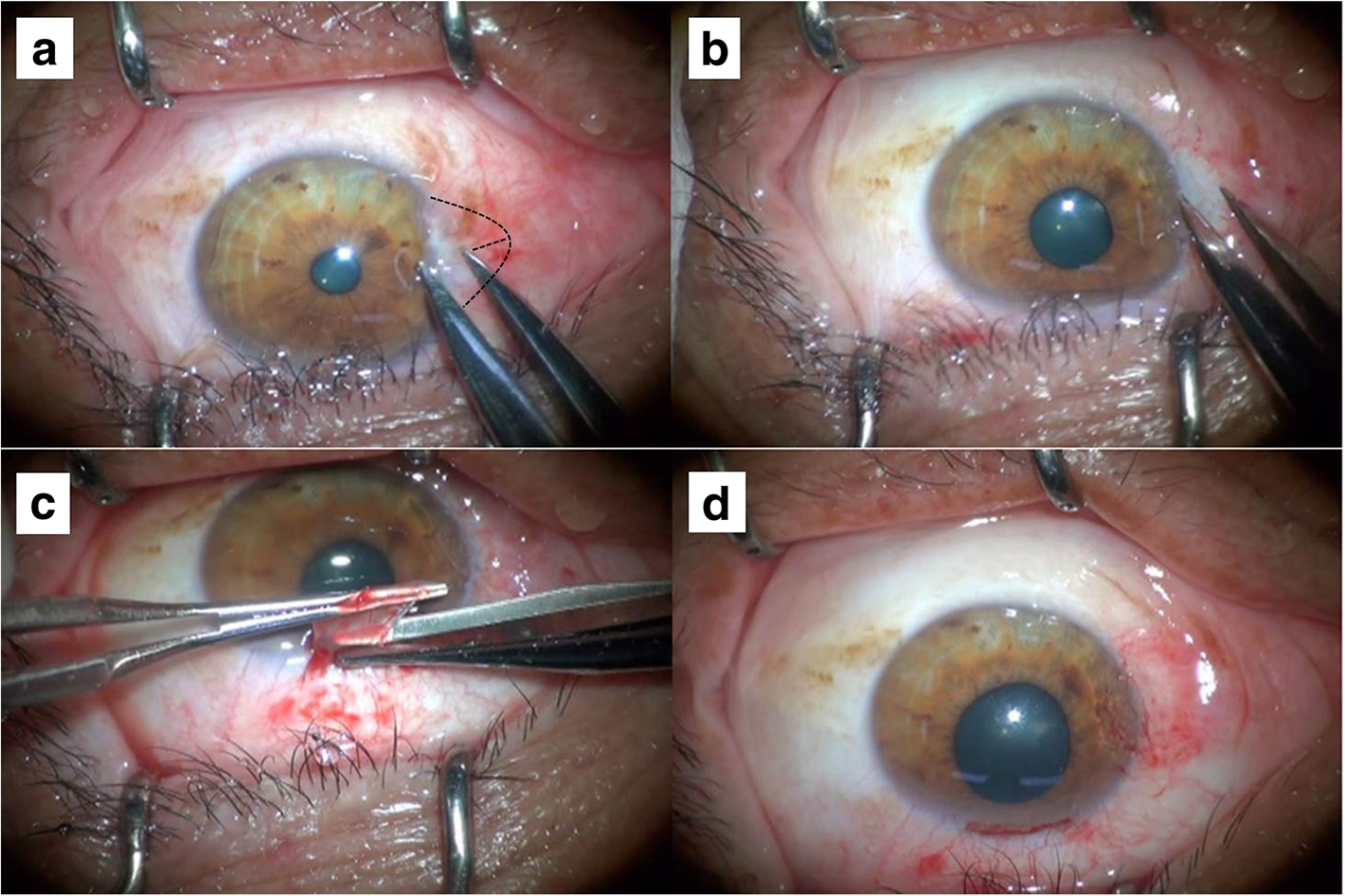
The length from the limbus to the apex of the pterygium head was measured (**a**). The head was removed with a #15 blade, the body and Tenon’s layer were excised with Vannas scissors, and the dimensions of the defect were measured (**b**). An autologous donor graft including limbal tissue was harvested from the superior bulbar conjunctiva (**c**). The graft was slid nasally with epithelium side down. Fibrin glue was placed on the defect and the graft is flipped epithelial side up with the limbal tissue facing the cornea (**d**)

Figures 1 and 2 are composites of photographs demonstrating the surgical procedure. The maximum length from the limbus to the apex of the pterygium head was first measured (Figs. 1b and 2a). Following the same line, a point with the same distance was marked from the limbus onto the pterygium body (Fig. 1c). From this point, two symmetric curvilinear lines were marked to the superior and inferior margins of the pterygium at the level of the limbus (Fig. 1c). A 2% subconjunctival lidocaine injection was administered at the pterygium head and body. After removing the head off the cornea with a #15 blade, the pterygium tissue within the curvlinear markings was excised along with Tenon’s layer using Vannas scissors (Fig. 2b). Hemostasis was achieved with minimal electrocautery. Intraoperative 0.2 mg/ml mitomycin C soaked sponges were applied to the bare sclera for 2 min followed by vigorous irrigation with BSS (Balanced Salt Solution, Alcon, Fort Worth, TX). Using a caliper, the dimensions of the defect were measured and the donor site on the superior bulbar conjunctiva were marked out with the same dimensions (Fig. 1d). After administering a 2% lidocaine subconjunctival injection, the donor conjunctiva was harvested with Vannas scissors. The graft included limbal tissue and excluded Tenon’s layer (Fig. 2c). The conjunctival autograft was secured using fibrin glue (Tisseel VH; Baxter, Vienna, Austria) as described by Koranyi et al [14]. The graft was placed with the epithelium side down on the cornea. The graft was carefully pushed to the nasal side with the limbal edge facing the wound. The two components of fibrin sealant were loaded separately in two syringes. One drop from each was placed over the recipient bed and the graft was quickly flipped over onto the pterygium defect and smoothed out (Fig. 2d). A bandage contact lens (Oasis, Johnson and Johnson: Jacksonville, FL) was placed on the cornea. Postoperatively, Moxifloxacin (Vigamox, Alcon, Fort Worth, TX) and 0.1% Fluorometholone (Ocumetholone, Samil, Seoul, Korea) were applied four times per day for 1 month. The contact lens was removed on postoperative day 3.

The patients were followed up on postoperative days 1, 14 and at 1, 3, 6 and 12 months after surgery.

Postoperative outcome data was recorded at 12 months after the surgery. All patients were followed up to at least 12 months postoperatively to monitor for any further changes.

## Results

Table 1 shows the baseline characteristics and outcomes of the 78 eyes from 78 patients (41 men and 37 women) included in this study. The mean age of the patients was 53.5 ± 14.2 (range 29–81) years. The mean length of the pterygium head was 1.9. ± 1.1 (range 0.7–5.1) mm. The mean duration of the operation was 20.9 ± 4.1 (range 15–30) minutes. The mean follow up period was 19.0 ± 4.9 (range, 14–30) months.

**Table 1:** Demographics, Operation Time, Follow-up Period, and Complications

Number of patients	78
Number of eyes	78
Age (years)	53.5 ± 14.2
Sex (M:F)	41:37
Horizontal length of pterygium (mm)^*^	1.9 ± 1.1
Operation time (min)	20.9 ± 4.1
Follow up period (months)	19.0 ± 4.9
Recurrence (%)	2 (2.6)
Undersized donor conjunctiva (%)	1 (1.3)
Oversized donor conjunctiva (%)	1 (1.3)
Subconjunctival hemorrhage (%)	7 (8.9)
Granuloma (%)	1 (1.3)
Conjunctiva injection at 4 wks (%)	2 (2.6)

^*^From limbus to apex of pterygium

**Fig. 3:**
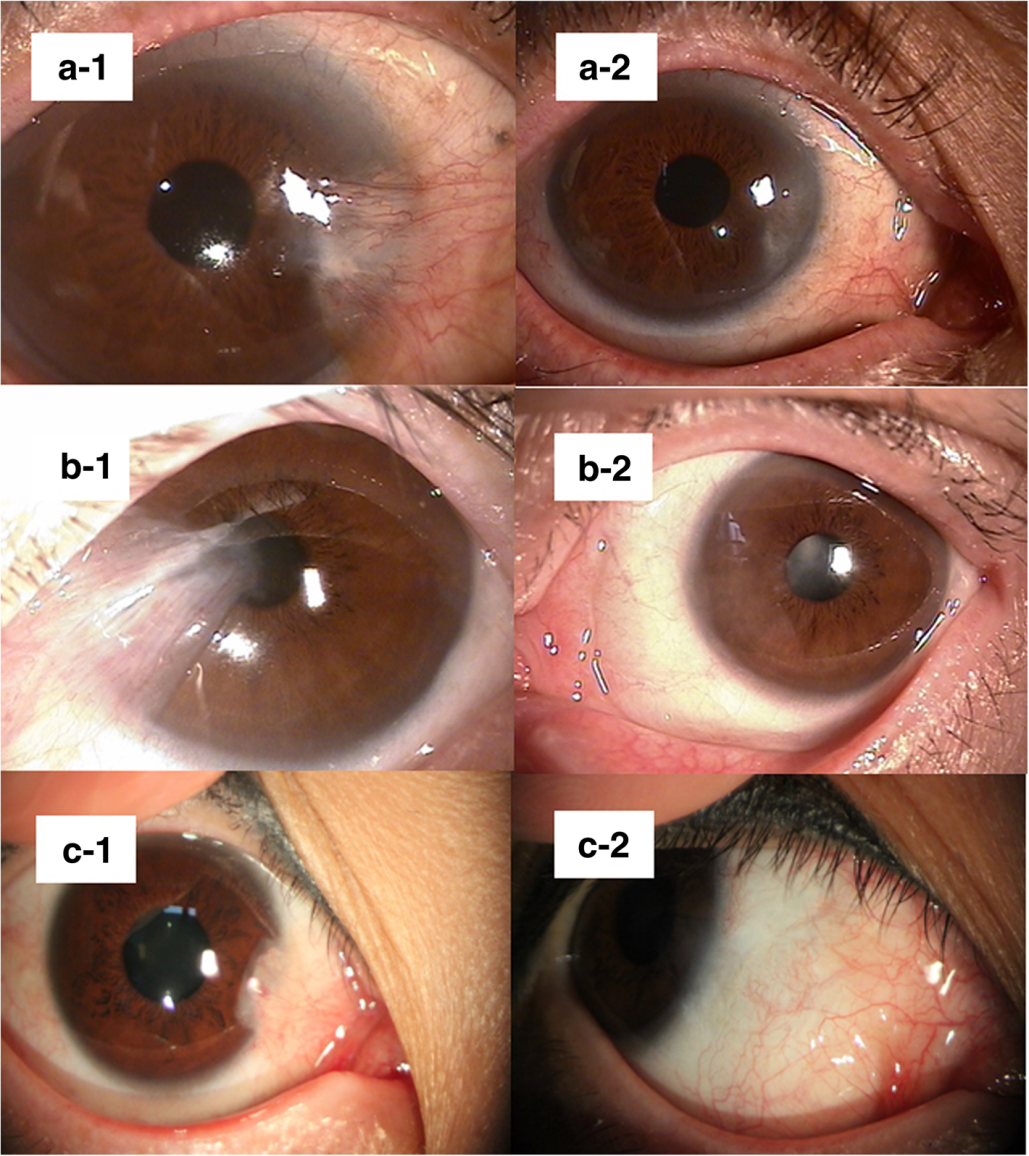
**A-1, B-1**, and **C-1** are preoperative images from three different patients. **A-2**, **B-2**, and **C-2** are images of respective outcomes

**Fig. 4:**
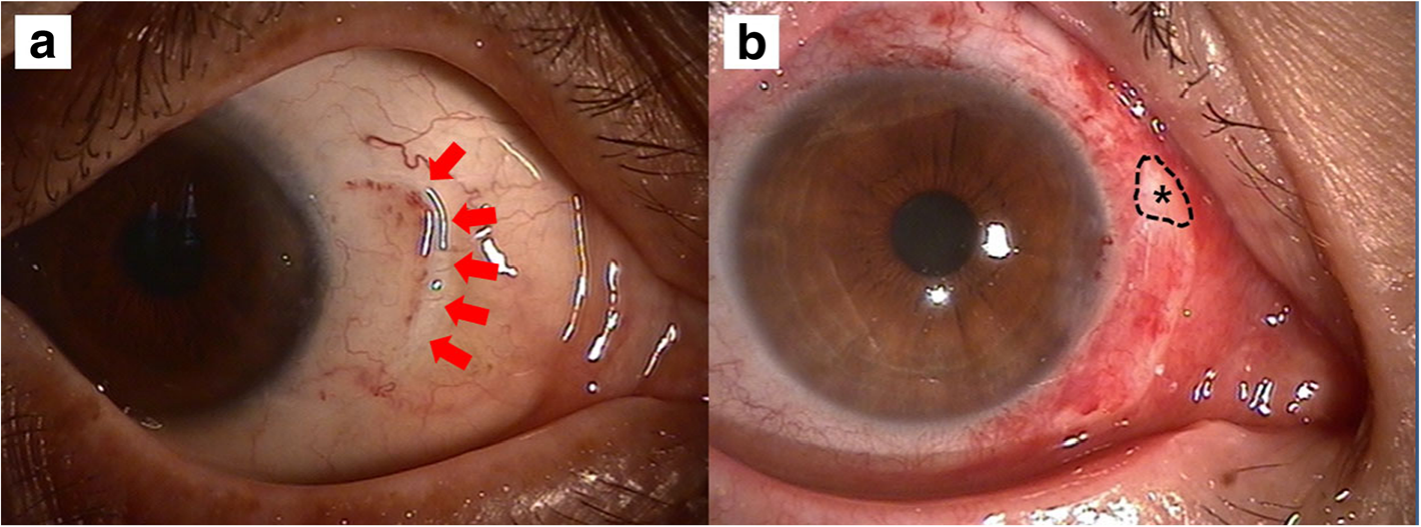
Two cases of inappropriately sized grafts. A small-sized donor conjunctiva with wound dehiscence, covered with conjunctiva marked with red arrows (**a**). An oversized donor conjunctiva with extraneous tissue marked with star (**b**)

Figure 3 is a composite of pre- and postoperative 12 month photographs of patients having satisfactory aesthetic outcomes. In these cases there was no sign of recurrence. The donor conjunctivae were well grafted and not injected. There was neither wound dehiscence due to undersized donor conjunctivae nor irregular surface due to oversized donor conjunctivae. There were only two cases of recurrence (2.6%). There were one case of undersized and one case of oversized graft, neither of which required surgical correction (Fig. 4). The undersized graft caused wound dehiscence and the oversized graft caused an uneven surface at the wound. But, there was no problem with regard to donor size in most of cases (97.4%). The most common complication was subconjunctival hemorrhage. There were seven cases of subconjunctival hemorrhage (8.9%), all resolving within 2 weeks. There was one case of granuloma formation (1.3%). There were two cases of conjunctival injection (2.6%) resolving by 2 months. None of these adverse outcomes occurred at the donor site on the superior bulbar conjunctiva.

## Discussion

The ideal pterygium surgery should use a simple technique as possible to optimize excellent aesthetics and minimize adverse outcomes. One reason our technique is simple is that we use fibrin glue instead of sutures. A meta-analysis comparing fibrin glue versus sutures in conjunctival autograft found fibrin glue was associated with shorter surgical times and a reduced recurrence rate [3]. Our mean operative time was 20.9 ± 4.1 min, comparable to other groups using fibrin glue. Ratnalingam et al. found that fibrin glue was associated with less postoperative pain [15]. The lower postoperative pain and reduced recurrence rate was hypothesized to result from less inflammation in the fibrin adhesive group. We did not use a standard measure to assess postoperative pain, but anecdotally our patients complained of very little discomfort in follow up visits.

Another simplification of our technique is that the excision is limited and does not extend to the posterior margin. At the present moment, there is no consensus regarding the optimal amount of excision that is necessary. Some argue for complete dissection and removal to the point of insertion, others argue for excision a certain distance from the limbus, and others argue for removal of just the pterygium head [7–12]. A more limited excision has a number of obvious advantages in that it should in theory require less anesthesia (subconjunctival as opposed to retro or peribulbar), shorten surgical time, and result in less postoperative pain. A major concern of limited excision techniques is the possibility of higher recurrence rates secondary to the residual pterygium left in place. It is therefore not considered a good option for more aggressive pterygia (e.g. recurrent pterygia, higher stage pterygia, etc). It is desirable to remove more pterygium tissue instead of exact length of pterygium expansion on the cornea in recurrent pterygia. For primary pterygia there is only one randomized control trial comparing recurrence rates and extent of excision with conjunctival autograft. Bazzazi et al. randomized 122 patients with primary pterygia to either conjunctival autograft with complete dissection and excision or resection of the pterygium head followed by placement of a small autograft. And they found no statistically significant difference in recurrence at 1 year [8]. Bahar et al. performed a conjunctival autograft cohort study of 161 patients with primary pterygia [7]. One group was a complete dissection and excision to the posterior margin and the other group was an excision limited to visible pterygium overlying conjunctiva. The study found no statistical difference in the total recurrence rate but found a statistically significant difference in the hazard ratio due to limited excision having earlier recurrences. With our limited excision technique for primary pterygia, we report a recurrence rate of 2.6%, a little less or comparable to recurrence rates reported for more extensive conjunctival autograft techniques on primary pterygia [16]. An additional benefit of our method is that it standardizes the geometry of the excision and graft and therefore should improve the quality of the graft fit. Of 78 cases, we only had two cases of incorrectly sized grafts, neither of which required surgical revision. Our review of the literature only showed one other method, the Stamp Technique, designed in particular to improve the fit of the graft [17].

A major difference in our technique compared to other limited excision conjunctival autograft techniques is that in ours the size of the excision is a precise function of the size of the pterygium head. The reasoning behind this approach is that in our experience pterygia with larger heads may be associated with more aggressively recurrent subtypes and therefore may benefit from relatively more extensive excision. Although we were unable to identify any studies investigating the direct association between the size of the pterygium head and recurrence rates, Tan et al. found a positive correlation between primary pterygium thickness and rate of recurrence [18]. Furthermore, Gazzard et al. found a correlation between pterygium thickness and the size of the pterygium head [19]. It therefore may follow that larger pterygium heads are associated with more aggressive subtypes and greater recurrence rates, and that these patients may benefit from more extensive excisions. There was no particular reason to select the exact length of pterygium expansion on the cornea as a measure for the excision of pterygium root. It is important that the size of the excision was a proportional to the size of the pterygium head in this study. So, it is possible to select half or double of the size of the pterygium head instead of same size.

We included the patients with only primary nasal pterygia in this study. We sometimes encountered temporal pterygia. We applied the same rule concerning the size of the excision. But it is necessary to compare the two groups (nasal versus temporal pterygia) concerning the size of the excision as a next study because the anatomy of the temporal conjunctiva is significantly different from that of the nasal conjunctiva.

A concern with any technique using MMC is toxic complications included but not limited to scleral melting, severe secondary glaucoma, and corneal edema [20, 21]. None of our cases resulted in complications associated with MMC. This may be because we used a low concentration (0.02%) on overall healthy eyes.

There are some limitations in our study. First, we did not have a control group to compare the effects of excision size, use of fibrin glue, and use of MMC. We therefore cannot analyze the effects of these different contributions had on our results. Second, our mean follow up period was 19.0 ± 4.9 months. It was once thought that 1 year follow up was sufficient to evaluate recurrence but new research has shown the risk to be continuous over the course of years [22]. Third, we measured the recurrence as a main surgical outcome, but we didn’t measure the more common outcome measures of appearance, irritation and vision (refractive error). Fourth, we designed pteryium excision size according to the length of pterygium (from limbus to apex) without considering the thickness of pterygium. Fifth, this study design is not prospective but retrospective. Sixth, three surgeons were involved in this study, so the study group is relatively heterogeneous.

## Conclusions

In conclusion, we proposed an optimal size of excision, intraoperative MMC and conjunctival autograft with fibrin glue in order to standardize graft sizes, simplify pterygium surgery, and reduce rates of incorrectly sized grafts. This technique showed excellent aesthetic outcomes and low rates of recurrence and other adverse events for primary pterygia.
